# Subjective dimensions of community participation following stroke: A qualitative approach

**DOI:** 10.6026/973206300223226

**Published:** 2026-06-30

**Authors:** Isha Akulwar-Tajane, Rajashree Naik

**Affiliations:** 1 Department of Physiotherapy, Lokmanya Tilak Municipal Medical College and Lokmanya Tilak Municipal General Hospital (LTMMC), Sion, Mumbai, Maharashtra, India &K J Somaiya College of Physiotherapy, Mumbai, Maharashtra, India; 2 Department of Physiotherapy, Physiotherapy Teaching and treatment center, Lokmanya Tilak Municipal Medical College and Lokmanya Tilak Municipal General Hospital (LTMMC), Sion, Mumbai, Maharashtra, India

**Keywords:** Community participation, stroke, subjective dimensions, patient-reported outcome measures, lived experience

## Abstract

Limited understanding of subjective dimensions of community participation remains a key gap in post-stroke rehabilitation. Therefore, it is of interest to explore lived experiences of 30 stroke survivors using cognitive interviews. Findings revealed reduced participation is mainly due to dependence, low self-efficacy and environmental barriers. Perceived social support and positive outcome expectations were identified as important facilitators of participation. Thus, we show the integration of subjective, patient-reported perspectives into rehabilitation assessment and intervention frameworks.

## Background:

Community reintegration is increasingly recognized as a central goal of rehabilitation for individuals with neurological conditions, particularly those recovering from stroke [1]. Beyond physical recovery, successful rehabilitation is measured by the ability of individuals to transition from basic household ambulation to effective community ambulation [2]. This transition reflects not only improved mobility but also the restoration of independence and participation in meaningful life roles. Community ambulation enables individuals to navigate real-world environments, engage in social interactions and resume occupational or recreational activities, all of which are essential for overall health and well-being [3]. Participation in employment, education and social roles contributes significantly to psychological stability, self-esteem and quality of life [4]. The importance of community mobility is strongly reflected in patient perspectives. Approximately 75% of stroke survivors consider the ability to get out and about in the community as essential or very important [5]. However, despite achieving adequate physical mobility in clinical settings, a substantial proportion of individuals nearly one-third remain unable to ambulate independently in the community. This limitation represents one of the most disabling long-term consequences of stroke. The inability to move freely in community settings often leads to reduced independence, increased reliance on caregivers and diminished opportunities for social engagement [6]. Community participation plays a crucial role in post-stroke recovery, extending beyond physical function to encompass social, emotional and psychological dimensions. Active engagement in community life has been associated with improved mental health outcomes, reduced social isolation and a lower risk of recurrent strokes. It allows individuals to reconnect with their social networks, access healthcare services and participate in activities that provide purpose and meaning [7]. Despite its importance, community participation remains one of the most persistent and under-addressed challenges in stroke rehabilitation. Evidence suggests that many stroke survivors do not return to their pre-strike levels of participation, even two to five years after the event. Limitations are observed across multiple domains, including employment, education, leisure activities and interpersonal relationships [8]. Although rehabilitation programs increasingly emphasize community reintegration, the concept of community participation itself remains complex and poorly defined. It is a multidimensional construct influenced by physical abilities, environmental barriers, personal motivation and social support systems [9]. Despite extensive research and numerous interventions aimed at improving participation outcomes, there is still no universally accepted definition or conceptual framework. This lack of clarity poses challenges in the development of standardized assessment tools and targeted rehabilitation strategies [10]. The term "participation" originates from the Latin words "pars" (part) and "capere" (to take), reflecting the idea of taking part in shared activities and social interactions. In contemporary healthcare, the most widely accepted definition is provided by the International Classification of Functioning, Disability and Health (ICF), which defines participation as "involvement in a life situation." The ICF framework highlights the dynamic interaction between personal and environmental factors and recognizes participation as a positive component of functioning [11]. Conversely, participation restrictions refer to difficulties an individual may experience in engaging in life situations. While the ICF has significantly influenced rehabilitation practices, overlaps between activity and participation domains remain a subject of ongoing debate, further emphasizing the need for clearer conceptualization [12]. To address this gap, the present study builds upon a prior scoping review that examined the construct and dimensions of community participation in neurological conditions. The study adopts a qualitative approach to explore how stroke survivors themselves perceive and experience community participation [13]. By focusing on lived experiences, the research aims to capture subjective dimensions that are often overlooked in quantitative assessments. Cognitive interviews, grounded in Husserl and phenomenology, were conducted to gain deeper insights into patients' perspectives. This approach allows for an in-depth understanding of how individuals interpret their participation within real-life contexts [14]. Therefore, it is of interest to report the subjective dimensions of community participation among stroke survivors, with a focus on their lived experiences, perceptions and psychosocial determinants influencing reintegration into community life.

## Methodology:

The present study adopted a qualitative research design grounded in phenomenology to explore the lived experiences and subjective dimensions of community participation among stroke survivors. A phenomenological approach was chosen to gain an in-depth understanding of how individuals perceive, interpret and construct their experiences following stroke, particularly in relation to participation in community life. Stroke survivors were recruited using convenience sampling from a physiotherapy outpatient rehabilitation unit of a tertiary care hospital. Participants were selected to ensure diversity in demographic and psychosocial characteristics, allowing for rich and varied data. Inclusion criteria comprised individuals aged between 18 and 70 years, diagnosed with stroke, living in the community (non-institutionalized) and ambulatory with or without assistive devices. Participants were required to have some level of experience with community participation post-stroke. Individuals with unstable medical conditions, concurrent neurological or psychiatric conditions, cognitive impairment, aphasia, psychiatric illness, or mobility limitations due to reasons other than stroke were excluded from the study. Ethical approval was obtained from the institutional ethics committee prior to the commencement of the study and all participants provided written informed consent. A-priori sample size was not calculated; and data collection was continued till data saturation was achieved, defined as the point where redundancy of information was seen *i.e.*, no new information was obtained with additional interviews. Data were collected using cognitive interviews conducted within a phenomenological framework, allowing participants to articulate their lived experiences in their own words. Interviews were conducted on a one-to-one basis, lasting approximately 60 to 120 minutes and were carried out in one or more sessions depending on participant comfort and fatigue levels. All interviews were audio-recorded with permission and later transcribed verbatim for analysis. While the primary source of information was the participant, limited input from caregivers was obtained where necessary for clarification. The interview guide was developed using theoretical constructs derived from the ICF and Social Cognitive Theory, focusing on domains such as self-efficacy, outcome expectations, self-regulation and social support. The phenomenological inquiry followed a hermeneutic (interpretive) approach, enabling the researcher to interpret and synthesize participants' experiences, emphasizing meaning- making and contextual understanding. Data analysis was conducted using Directed Qualitative Content Analysis (DQICA). A preliminary coding framework (codebook) was developed a priori based on existing literature and theoretical models. Primary codes included constructs such as self-efficacy, outcome expectations and social support. During the analysis, secondary codes emerged inductively from participant narratives, allowing refinement and expansion of themes. Transcripts were managed and analyzed using *in vivo* version 12 software (QSR International Pty Ltd.) to facilitate systematic organization and coding of data. The analysis focused on identifying recurring patterns, key themes and relationships across participants, thereby providing a comprehensive understanding of subjective community participation post-stroke.

## Results:

Data was collected for a total of 30 stroke survivors after which no new themes or insights emerged from additional interviews. One of the major inclusion criteria was an experience in community participation post-stroke. Participants were recruited at unspecified times after stroke regardless of their current level of participation in the community. Heterogeneity with respect to psychosocial factors and diversity in demographics was intended for the richness of the data and is seen in this sample. Their demographic characteristics are displayed in Table 1. ICF and Social cognitive theory constructs were used as guiding frameworks to structure the questionnaire for the interview and for the data analysis. In depth conversations were conducted on a one-to-one basis for 60-120 minutes divided into one or more sessions; and were audio recorded for later use. The majority of the information was taken from the patients themselves with only some information obtained or confirmed from the caregiver as appropriate. Qualitative interviews were conducted using Heidegger's approach of phenomenology (also labelled as interpretive or hermeneutic phenomenology). The primary investigator sought to identify the essential structures of phenomena of interest focusing on existential themes, their day-to-day experience of community participation, that relate to living with stroke. Feelings, thoughts and perceptions they derive were captured in a descriptive language and noted in a detailed and narrative manner from the first-person perspective. Though the primary focus of inquiry was on the participant's own experience, a detailed description and creative synthesis of the experience was co-created with relational dynamics between the researcher and participant generated through the research dialogue. The researcher identified and articulated the essential structures of lived experience to develop a shared understanding across participants. Audio-recordings were transcribed into intelligent verbatims as suitable for thematic analysis. NVivo version 12 software (QSR International Pty Ltd; Chatstone, Victoria, Australia) was used to organize the data. Interview transcripts were analyzed using Deductive/Directed Qualitative Content Analysis (DQICA). The directed content analysis approach is a deductive qualitative research method that uses existing theories, frameworks, or research to guide the coding and analysis of data. Instead of generating themes from the data, this structured approach starts with predefined categories to test, confirm, extend, or replicate previous findings. Researchers apply these predetermined codes to their textual or visual data, allowing for a focused examination of specific concepts and relationships within the content. This step is used to extend the application of the theory/ies to contexts/cultures other than those in which that/those theory/ies was/were developed. In the present study, a codebook of Primary codes was created a-priori, based on the literature, predominantly guided by Social Cognitive Theory to investigate the constructs viz. Outcome expectations, self-efficacy, self-regulation &social support, etc. for community participation. Secondary codes were created during the analytic process to identify emergent themes within each primary code from participants' responses. The key themes and patterns emerged from consistent findings across participants. The majority of the participants experienced participation restrictions in daily activities, mobility, social roles, employment and economic self-sufficiency compared to their pre-stroke participation. They reported low satisfaction with participation majorly because of dependence on others; and independence and recovery were perceived as important outcome expectations. Participants consistently reported low self-efficacy but positive outcome expectations for community participation, while reports for self-regulation and social reports were much more variable. Low self-efficacy was majorly related to their concern about physical limitations especially the movement control of the lower limb, magnitude of which was higher for community walking, because of uncertainty of external environment &delay in initiating motor responses. Whereas, a person's confidence in their capability to manage and participate in life activities, even with limitations, strongly correlates with higher participation. Self-efficacy was influenced by mental perception and mastery experiences; and was enhanced through praise and encouragement by family members. Self-motivation &Positive thinking strengthened the self-efficacy with more involvement in physical activities &social participation. Satisfaction with the living environment was a determinant of participation. Self-regulation was not very evident as participants showed less active decision-making abilities. Age, Gender &Employment status brought confounding influences and thus variability in responses. Directly or indirectly a feeling of social isolation and reduced well-being was evident.

## Discussion:

Among the environmental factors, social support, relationships and attitude towards patients appeared to be major determinants of participation as well as physical environment and societal environment (access to health, social services and policies). We found that a person's home environment was related to one's level of participation in ADLs, particularly in the period shortly after discharge from an acute care or inpatient rehabilitation hospital [15]. Though this finding is not surprising, it does provide empirical evidence that supports the commonly held belief that participation in life activities is associated, at least in part, with aspects of the environment in which a person lives. Physical factors within the environment, however, might not be as important in the long term, as people adapt to their situations [16]. In this study, social support was associated with greater social and home participation 6 months after discharge from an acute or inpatient rehabilitation hospital, underscoring the importance of social environment in the long run. Social support may impact one's ability to overcome the environmental challenges outside of the home in the setting of a physical impairment and may also act as a protective factor against post-stroke depression. Social support provided physical assistance, but was also perceived as a barrier when family &friends were overprotective [17]. Among the psychosocial factors, immediate family and social support stand out to relatively more weightage received from the participants as an enabler or disabler to their participation. Social support can come from a wide range of sources such as family, friends, significant others, social networks, religious organizations, or community groups. Social support can also be the actual assistance a person receives from others or the perceived support that results from the confidence of the availability of support for physical or emotional needs [18]. Social support is an environmental factor that has been shown to be a positive prognostic indicator for physical and psychological outcomes after stroke. However, there is limited evidence in the literature to suggest the existence of a positive relationship between social support and participation [19]. Also, this relationship may differ as per the cultural norms, beliefs and expectations; and thus, it depends upon the individual's geographical context. Given the limited evidence in general and the cultural and health system differences amongst countries, there is a need to further elucidate the association between participation and the perceived social support after stroke in future studies [20]. Recent study on contextual determinants of post-stroke participation also identified social support, people's attitudes, physical environment, access to health and social services, policies and environmental factors as major facilitators in post-stroke participation. The findings of our study align well with documented lived experience of people with stroke and the disability community in general, suggesting the importance of rehabilitation interventions focusing on navigating environmental barriers (*e.g.*, use of community resources and services, peer navigators, advocacy training). Most of these factors are modifiable and should be addressed to improve post-stroke participation [21]. Personal factors, particularly psychological and psychosocial factors, were identified as positively associated with post-stroke participation. Psychological factors include motivational aspects, acceptance of a new condition, self-esteem, etc., in a recent review by Vecchia *et al.* (2021) [22], socio-demographic determinants were mostly unrelated with participation or showed discordant and inconclusive results. Although less investigated, psychosocial/psychological factors, particularly self-esteem and acceptance, were associated with participation in most studies. Motivation was found in qualitative studies, but discordant in quantitative ones. Also, in previous studies, perceived recovery is identified as a predictor of participation in leisure and outdoor activities [23]. This highlights the importance of considering non-neurologic clinical assessments that include patients' self-evaluations. Given that perceived recovery is a modifiable psychological construct, future research may consider whether intervention directed at increasing self-efficacy, resilience and perceived recovery may improve participation outcomes. Interventions focusing on social model of disabilities could positively influence patients' perceptions of their recovery after a stroke. A structured evaluation of determinants of participation may be used in clinical practice to propose appropriate support and then improve patients' recovery [24].

## Conclusion:

Exploring subjective experiences of community participation is essential to better understand post-stroke recovery. Meaningful engagement improves quality of life and supports a patient-centred, biopsychosocial approach. Self-efficacy and social support are key factors, highlighting the importance of incorporating patients' perspectives.

## Data availability:

The interview questionnaire and filled sample forms are available upon request to the corresponding author.

## Abbreviations:

ICIDH: International Classification of Impairments, Handicap and Disability

ICF: International Classification of Functioning, Disability and Health

## Figures and Tables

**Table 1:** Demographic characteristics of the participants

**n = 30**	**Mean ± SD**	**Range**
Age (in years)	54 ± 7.6	34 - 69
Sex	Males (86.66%)	Females (23.34%)
Duration of stroke (in-months)	13 ± 6.3	26-Jun
Living status	Home (100%)	
Marital status	Married (80%)	Unmarried (20%)
Employment status	Employed &Working (6.66%)	
	Employed &N/ot working (13.33%)	
	Retired (80%)	
